# Editorial: Non-invasive stimulation: role in neurorehabilitation

**DOI:** 10.3389/fresc.2023.1263530

**Published:** 2023-08-08

**Authors:** Naaz Desai (Kapadia), Cesar Marquez-Chin, Robert Chen

**Affiliations:** ^1^ Division of Brain, Imaging and Behaviour, Krembil Brain Institute, Toronto, ON, Canada; ^2^ CRANIA, University Health Network and University of Toronto, Toronto, ON, Canada; ^3^ The KITE Research Institute, Toronto Rehabilitation Institute—University Health Network, Toronto, ON, Canada; ^4^ Institute of Biomedical Engineering, University of Toronto, Toronto, ON, Canada; ^5^ Edmond J. Safra Program in Parkinson’s Disease, Morton and Gloria Shulman Movement Disorders Clinic, Toronto Western Hospital, UHN, Toronto, ON, Canada; ^6^ Divison of Neurology, Department of Medicine, University of Toronto, Toronto, ON, Canada; ^7^ Institute of Medical Science, University of Toronto, Toronto, ON, Canada

**Keywords:** noninvasive brain stimulation, neuromuscular stimulation, stroke, spinal cord injury, rehabilitation, neuromodulation

**Editorial on the Research Topic** Non-invasive stimulation: role in neurorehabilitation

For many acquired chronic neurological conditions, rehabilitation remains the most promising treatment. In the past few decades, with the evolving understanding of neuroplasticity, researchers have investigated various therapeutic modalities that taps into these mechanisms to improve patient outcomes. Whereas both peripheral and central stimulation techniques have been pursued, only recently have researchers applied a combination of these techniques to improve motor outcomes, reduce therapy duration, or both. In this special topic, we compiled articles that used various non-invasive stimulation techniques to understand and promote motor recovery in different neurological conditions including stroke, spinal cord injury, traumatic brain injury, Parkinson’s disease and multiple sclerosis.

Non-invasive peripheral stimulation techniques, including functional electrical stimulation, sensory stimulation, electrical muscle stimulation and transcutaneous electrical stimulation, are some of the classical neuromodulation modalities therapists use for neurorehabilitation (1–4). Although these techniques have shown promise, the literature shows that the results are highly variable (5). Therefore, there is an imminent need to develop treatment modalities that can consistently produce good outcomes. In one such attempt, rehabilitation interventions combining peripheral stimulation with central stimulation are being actively studied. Stefan et al., showed that an enduring change in excitability in the cortical output circuitry can be induced in the human motor cortex by the conjoint activity of somatosensory afferents and intrinsic motor cortical circuits (6). Liu et al., proposed that central intervention and peripheral intervention maybe combined to form closed-loop information feedback to enhance brain plasticity and remodeling of neural pathways which may result in improved performance or outcomes (7). Common noninvasive brain and spinal cord stimulation techniques that have been used in this regard include but are not limited to transcranial magnetic stimulation (TMS), transcranial direct current stimulation (tDCS), transcutaneous spinal cord stimulation (tSCS) and transcranial focused ultrasound (TUS) (8–10). Besides, scientists have also used some of the above techniques to predict recovery profiles in conditions such as traumatic spinal cord injuries.

This research topic brings together an exciting compilation of manuscripts that have applied non- invasive stimulation techniques to further the diagnosis and treatment of various neurological disorders. Bersch-Porada et al. discussed the use of motor point mapping in addition to standardized measures such as International Standards for Neurological Classification of Spinal Cord Injury (ISNCSCI) and manual muscle test as possible predictors of motor recovery and proposed the use of motor point mapping for developing individualized treatment plans in patients with traumatic cervical spinal cord injuries. Arora et al. conducted a review of TMS-based measures that may aid better prognostication and advance the understanding of the neurophysiologic mechanisms underlying impairments and functional recovery in spinal cord injury. Under the interventional realm, the first manuscript is a case study by McGeady et al. which explored the benefits of brain computer interface (BCI) motor priming prior to delivery of tSCS compared to tSCS training alone in improving upper extremity motor function. The second manuscript by Foglia et al. studied the efficacy and safety of 10 Hz repetitive TMS (rTMS) in a spinal cord injury patient on intrathecal baclofen pump therapy presenting with drug resistant neuropathic pain. The next article, by Anderson et al. assessed the effectiveness of functional electrical stimulation therapy of the upper extremities delivered using a transcutaneous multi-channel stimulator called the MyndMove in individuals with cervical spinal cord injury. The last manuscript is a review by Cortez-Grippe et al., which illustrated the central and peripheral neurostimulation protocols that have been used in the treatment of functional movement disorders and discussed the efficacy, limitations, and possible future clinical applications of these techniques.

This collection emphasized the potential of non-invasive neurostimulation techniques in both the diagnostic and therapeutic domains. It also highlights the need for further research in the emerging area of combination neurostimulation therapies, which have the potential to transform care for individuals living with the devastating effects of chronic neurological conditions.
